# Supplementary material for: OscoNet: inferring oscillatory gene networks
Source: BMC Bioinformatics. 2020 Aug 21;21(Suppl 10):351. doi: 10.1186/s12859-020-03561-y (PMC7445923; doi:10.1186/s12859-020-03561-y)
Supplement: Supplementary file 1 — Additional file 1 A supplementary report is available (supplementary.pdf). [file 12859_2020_3561_MOESM1_ESM.pdf]

Supplementary material for 'OscoNet: Inferring oscillatory gene networks'

Alexis Boukouvalas, Luisa Cutillo, Elli Marinopoulou,
Nancy Papalopulu and Magnus Rattray

April 11, 2019

Contents

1 Synthetic data **1**

2 Microarray data **2**

3 Single cell H1 bootstrap NULL distributions **2**

4 Oscope pairwise distance metric **2**

1 Synthetic data

In Figure 1 we show the FDR achieved by standard Oscope and the bootstrap hypothesis test. In all cases, the bootstrap hypothesis test improves as the number of samples is increased. Standard Oscope was examined at levels $[0., 0.05, 0.11, 0.16, 0.21, 0.26, 0.32, 0.37, 0.42, 0.47, 0.53, 0.58, 0.63, 0.68, 0.74, 0.79, 0.84, 0.89, 0.95, 1.]$. In Table 1 we show the FDR, TPR and FPR achieved by Oscope at the default 5% threshold level and the OscoNet bootstrap test run with 2000 samples across all noise levels used. The Oscope method consistently achieves a perfect TPR (1.00) but the number of false positives increases for higher noise levels. In contrast the OscoNet method, achieves lower TPR as the noise level increases but maintains a low level of false positives (FPR).

Method	FDR	TPR	FPR
Noise 0.05			
Oscope	0.91	1.00	0.100
OscoNet B=2000	0.09	1.00	0.001
Noise 0.1			
Oscope	0.92	1.00	0.130
OscoNet B=2000	0.08	1.00	$< 1e-3$
Noise 0.2			
Oscope	0.95	1.00	0.210
OscoNet B=2000	0.09	0.93	$< 1e-3$
Noise 0.3			
Oscope	0.95	1.00	0.210
OscoNet B=2000	0.06	0.73	$< 1e-3$
Noise 0.4			
Oscope	0.97	1.00	0.376
OscoNet B=2000	0.07	0.57	$< 1e-3$

Table 1: Synthetic data. False discovery rate (FDR), true positive rate (TPR) and false positive rate (FPR) for the Oscope method run with the default 5% threshold and the OscoNet hypothesis test with $B = 2000$ samples.

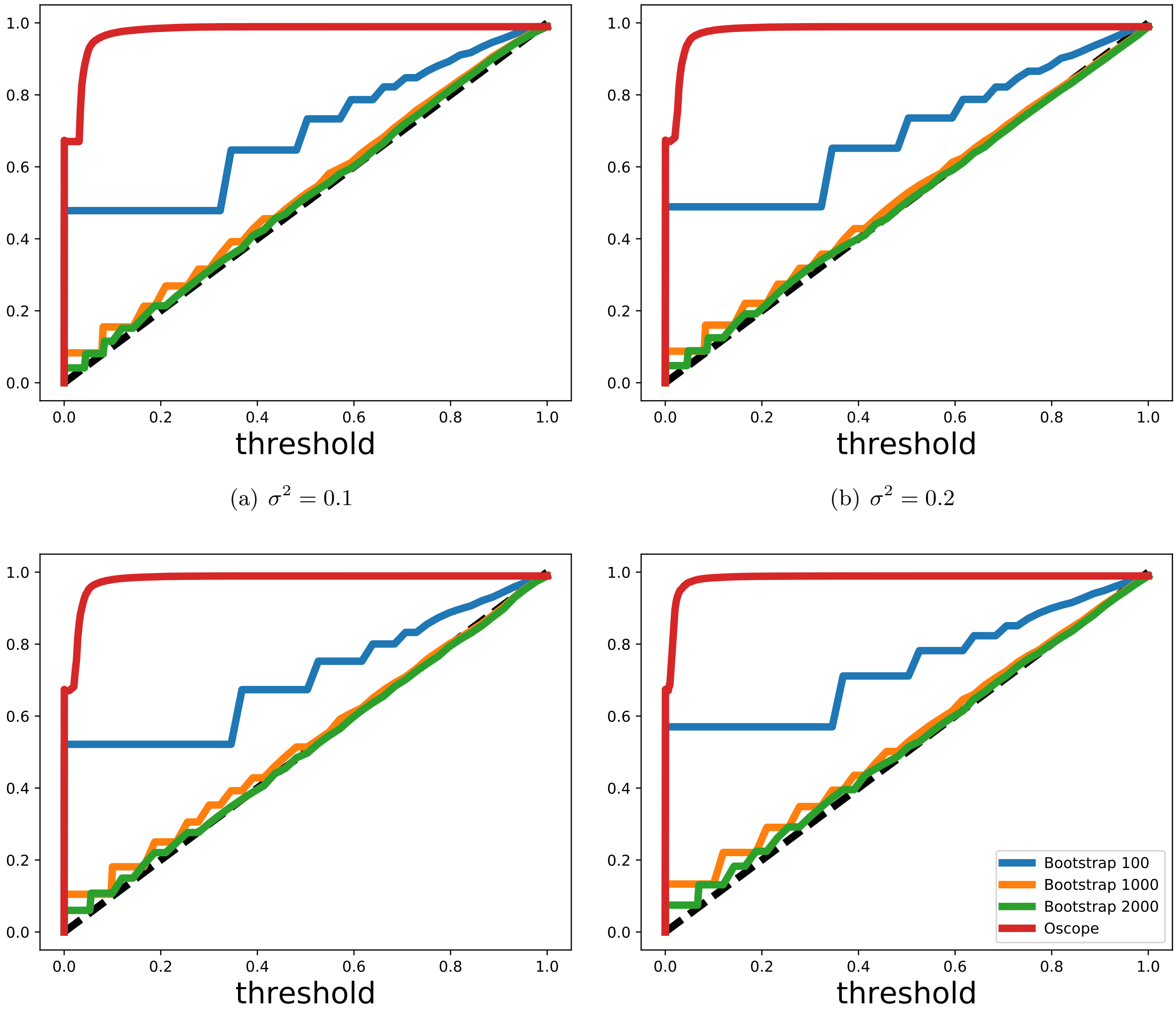

(a) $\sigma^2 = 0.1$

(b) $\sigma^2 = 0.2$

(c) $\sigma^2 = 0.3$

(d) $\sigma^2 = 0.4$

Figure 1: False discovery rate for the synthetic study for noise levels $\sigma^2 = [0.1, 0.2, 0.3, 0.4]$. The FDR rate (vertical axis) plotted again the different threshold levels (horizontal axis) for each method.

2 Microarray data

The peak time summaries are confirmed in Figure 2 where for each of 72 genes in the Oscope cluster, we plot the estimated and true peak times for each pseudotime method; the ENI estimates have little correspondence to the true times whereas the spectral method accurately predicts the peak times for most genes. The spectral method's skill improves when using the larger OscoNet 265 gene set.

3 Single cell H1 bootstrap NULL distributions

We show the NULL distributions for two genes in Figure 3. The NULL distribution is very different for the two pairs even though the KPNA2 gene participates in both pairs. For the KPNA2-LPIN3 pair, there is no evidence of co-oscillation in contrast to the KPNA2-CCNB1 pair.

4 Oscope pairwise distance metric

We rederive the pairwise distance from Leng *et al.* (2015) to clarify its properties.

Let two genes be $X = \sin(\omega t + \phi)$ and $Y = \sin(\omega t + \phi + \Psi)$, that is they have identical profiles except for

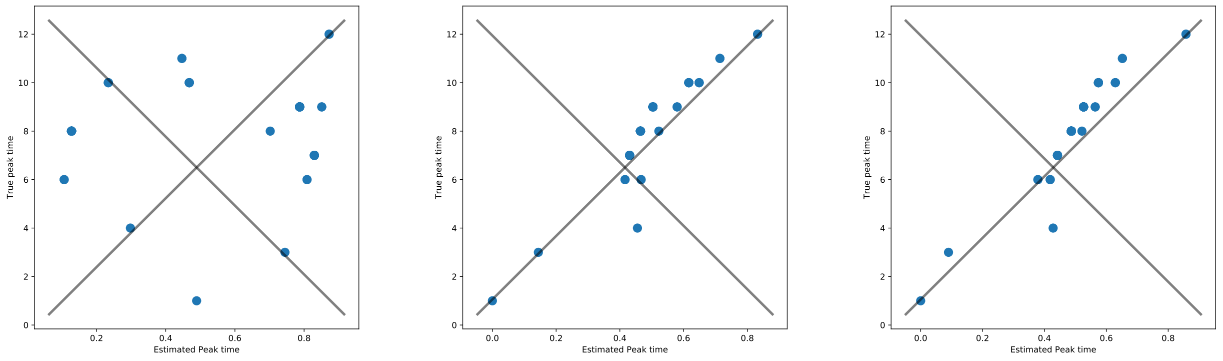

(a) ENI trained on 72 genes $R = 0.17$ (b) Spectral trained on 72 genes $R = 0.94$ (c) Spectral trained on 265 genes $R = 0.99$

Figure 2: Microarray data: Cell cycle peak time correlation reported for each algorithm. The Spearman correlation is also reported (R).

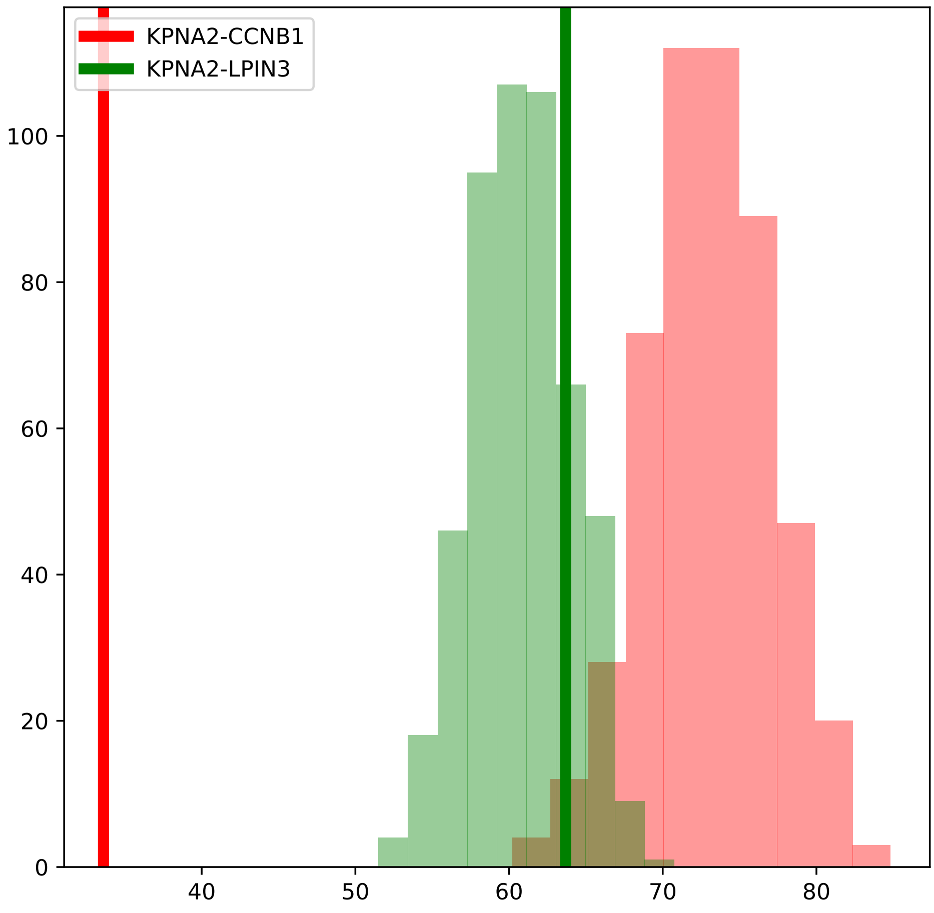

Figure 3: Single cell H1 data: Null distributions (histograms) arising from permuting the cell order and unpermuted p-value (vertical bars) for two pairs of genes.

a a phase shift Ψ.

$$
\begin{aligned}
Y &= \sin(\omega t + \phi + \Psi) = \sin(\omega t + \phi)\cos(\Psi) + \cos(\omega t + \phi)\sin(\Psi) \\
&= X\cos(\Psi) \pm \sqrt{1 - X^2}\sin(\Psi)
\end{aligned}
$$

Square both sides

$$(Y - X\cos(\Psi))^2 = \left(1 - X^2\right)\sin^2(\Psi)$$

Rearranging

$$
\begin{aligned}
Y^2 + X^2\left[cos^2(\Psi) + \sin^2(\Psi)\right] - 2YX\cos(\Psi) - \sin^2(\Psi) = 0 \\
Y^2 + X^2 - 2YX\cos(\Psi) - \sin^2(\Psi) = 0
\end{aligned}
$$

Assuming the the cell population is homogeneous, i.e. the functions are the same (same angular velocity ω, starting phase ϕ), we can then compute the pairwise distance between any two genes given their phase shift:

$$d(X, Y|\Psi) = \sum_{s=1}^{N}\left[Y_s^2 + X_s^2 - 2Y_sX_s\cos(\Psi_{XY}) - \sin^2(\Psi_{XY})\right]$$

for N cells.

To estimate the phase shift, the pairwise gene distance is minimized with respect to Ψ:

$$\widehat{\Psi} = \arg\min_{\Psi}\ d(X, Y|\Psi)$$

References

Leng, N., Chu, L.-F., Barry, C., Li, Y., Choi, J., Li, X., Jiang, P., Stewart, R. M., Thomson, J. A., and Kendziorski, C. (2015). Oscope identifies oscillatory genes in unsynchronized single-cell rna-seq experiments. *Nature methods*, **12**(10), 947–950.
